# Leukocyte telomere length in paediatric critical illness: effect of early parenteral nutrition

**DOI:** 10.1186/s13054-018-1972-6

**Published:** 2018-02-21

**Authors:** Sören Verstraete, Ilse Vanhorebeek, Esther van Puffelen, Inge Derese, Catherine Ingels, Sascha C. Verbruggen, Pieter J. Wouters, Koen F. Joosten, Jan Hanot, Gonzalo G. Guerra, Dirk Vlasselaers, Jue Lin, Greet Van den Berghe

**Affiliations:** 1 Clinical Division and Laboratory of Intensive Care Medicine, Department of Cellular and Molecular Medicine, Herestraat 49, B-3000 Leuven, Belgium; 2 Intensive Care Unit, Department of Paediatrics and Paediatric Surgery, Erasmus Medical Centre, Sophia Children’s Hospital, Rotterdam, The Netherlands; 3 Department of Paediatrics, Intensive Care Unit, University of Alberta, Stollery Children’s Hospital, Edmonton, Canada; 4 Department of Biochemistry and Biophysics, University of California, San Francisco, CA USA

**Keywords:** PICU, Critical illness, Critical care, Intensive care, Telomeres, Telomere length, Nutrition, Paediatric, Children

## Abstract

**Background:**

Children who have suffered from critical illnesses that required treatment in a paediatric intensive care unit (PICU) have long-term physical and neurodevelopmental impairments. The mechanisms underlying this legacy remain largely unknown. In patients suffering from chronic diseases hallmarked by inflammation and oxidative stress, poor long-term outcome has been associated with shorter telomeres. Shortened telomeres have also been reported to result from excessive food consumption and/or unhealthy nutrition. We investigated whether critically ill children admitted to the PICU have shorter-than-normal telomeres, and whether early parenteral nutrition (PN) independently affects telomere length when adjusting for known determinants of telomere length.

**Methods:**

Telomere length was quantified in leukocyte DNA from 342 healthy children and from 1148 patients who had been enrolled in the multicenter, randomised controlled trial (RCT), PEPaNIC. These patients were randomly allocated to initiation of PN within 24 h (early PN) or to withholding PN for one week in PICU (late PN). The impact of early PN versus late PN on the change in telomere length from the first to last PICU-day was investigated with multivariable linear regression analyses.

**Results:**

Leukocyte telomeres were 6% shorter than normal upon PICU admission (median 1.625 (IQR 1.446–1.825) telomere/single-copy-gene ratio (T/S) units vs. 1.727 (1.547–1.915) T/S-units in healthy children (*P* < 0.0001)). Adjusted for potential baseline determinants and leukocyte composition, early PN was associated with telomere shortening during PICU stay as compared with late PN (estimate early versus late PN –0.021 T/S-units, 95% CI −0.038; 0.004, *P* = 0.01). Other independent determinants of telomere length identified in this model were age, gender, baseline telomere length and fraction of neutrophils in the sample from which the DNA was extracted. Telomere shortening with early PN was independent of post-randomisation factors affected by early PN, including longer length of PICU stay, larger amounts of insulin and higher risk of infection.

**Conclusions:**

Shorter than normal leukocyte telomeres are present in critically ill children admitted to the PICU. Early initiation of PN further shortened telomeres, an effect that was independent of other determinants. Whether such telomere-shortening predisposes to long-term consequences of paediatric critical illness should be further investigated in a prospective follow-up study.

**Trial registration:**

ClinicalTrials.gov, NCT01536275. Registered on 16 February 2012.

**Electronic supplementary material:**

The online version of this article (10.1186/s13054-018-1972-6) contains supplementary material, which is available to authorized users.

## Background

Survival of children who require treatment in the paediatric intensive care unit (PICU) for life-threatening diseases, extensive surgery, or trauma has improved [1, 2]. However, PICU survivors continue to suffer from an important long-term legacy of critical illness, characterised by impaired physical and neurocognitive development [3]. This legacy could be explained by the pre-admission disease [4] or be induced or aggravated by the acute event and/or the intensive medical care. Whether alterations in telomere length could play a role has not been investigated.

Telomeres are nucleoprotein complexes at the termini of eukaryote chromosomes that protect chromosome ends from degradation and prevent fusion with neighbouring chromosomes [5]. Telomeres shorten with each cell cycle [6], making them good markers of overall cellular replicative capacity and senescence [7]. This telomere shortening process can be accelerated by environmental and lifestyle factors, many of which increase oxidative stress levels and inflammation, such as smoking, life stress and exposure to air pollution [7–9]. Furthermore, shorter telomeres have been reported in leukocyte DNA from children with chronic medical conditions [10, 11], possibly due to more cell replication cycles in immune cells, which has been associated with long-term metabolic and cardiovascular morbidity and mortality [12, 13]. Interestingly, evidence suggests that macronutrient restriction and/or healthy feeding habits may slow down the progressive shortening of telomeres, which could protect against age-related diseases [14, 15].

Whether critically ill children are admitted to the PICU with shorter telomeres reflecting the premorbid state is unknown. Also, it remains unknown whether the use of parenteral nutrition (PN) early during critical illness in children, or instead accepting a substantial macronutrient deficit when only relying on enteral feeds that often fail in such patients, affects leukocyte telomere length.

As critically ill children often suffer from an underlying chronic disease or are admitted to the PICU after an insult characterised by hypoxia or inflammation, we hypothesised that leukocyte telomeres in critically ill children upon PICU admission are shorter than those of matched healthy children. Also, as previous studies performed in rodent models and in disease-free adults have suggested shortened telomeres as the result of excessive food consumption [14, 15], we further hypothesised that initiation of supplemental PN given early during critical illness, as compared with not using PN for one week in the PICU, an intervention that has been shown to slow down rather than accelerate recovery, could further shorten telomeres by the time of PICU discharge, directly or indirectly via its slowing effect on recovery [2, 16].

## Methods

### Study population

**Fig. 1:**
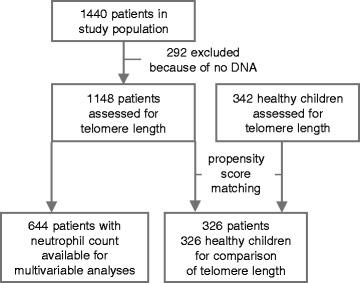
Consolidated Standards of Reporting Trials (CONSORT) flow diagram of the study participants

This pre-planned secondary analysis included 1148 critically ill children (0–17 years), enrolled in a randomised controlled trial on macronutrient management in PICU (PEPaNIC-study, *N* = 1440) [2] and 342 healthy children, from whom leukocyte DNA was available (Fig. 1, Table 1). Patients had been randomised to early initiation of PN supplementing insufficient enteral nutrition to reach the caloric target within 48 h (early PN), or to postponing any PN to beyond the first week in PICU if enteral nutrition was still insufficient (late PN).

**Table 1:** Demographics

	Total population	Subgroup with neutrophil count available	Matched subgroups^a^	
Baseline characteristics	PICU patients *N* = 1148	PICU patients *N* = 644	PICU patients *N* = 326	Healthy children *N* = 326
Male sex, *N* (%)	640 (55.7)	355 (55.1)	180 (55.2)	179 (54.9)
Age (years)	1.8 (0.3–7.4)	1.6 (0.3–5.9)	4.5 (1.7–8.7)	4.5 (1.8–7.8)
Age <1 year, *N* (%)	476 (41.5)	276 (42.9)	51 (15.6)	44 (13.5)
Height (%)^b^	38.7 (8.5–78.2)	35.1 (7.8–71.5)	38.1 (8.5–78.4)	
Weight (%)^b^	32.5 (8.3–67.8)	29.8 (6.5–62.2)	39.8 (14.8–70.0)	
Chronicity, *N* (%)^c^	882 (76.8)	532 (82.6)	242 (74.2)	
Randomisation to late PN, *N* (%)	576 (50.2)	324 (50.3)	167 (51.2)	
STRONGkids risk level, *N* (%)^d^				
Medium	1046 (91.1)	605 (93.9)	299 (91.7)	
High	102 (8.9)	39 (6.1)	27 (8.3)	
PeLOD score first 24 h^e^	22 (12–32)	31 (21–32)	21 (11–31)	
PIM2 score^f^	−2.7 (−3.6; –1.4)	−3.0 (−3.7; –1.7)	−2.7 (−3.8; –1.5)	
PIM2 probability of death (%)^g^	6.3 (2.6–19.3)	5.0 (2.4–15.5)	6.5 (2.6–22.0)	
Emergency admission, *N* (%)	554 (48.3)	218 (33.9)	159 (48.8)	
Diagnostic category, *N* (%)				
Surgical				
Abdominal	51 (4.4)	19 (3.0)	7 (2.2)	
Burns	8 (0.7)	1 (0.2)	4 (1.2)	
Cardiac	517 (45.0)	377 (58.5)	128 (39.3)	
Neurosurgery-traumatic brain injury	106 (9.2)	51 (7.9)	40 (12.3)	
Thoracic	46 (4.0)	29 (4.5)	11 (3.4)	
Transplantation	22 (1.9)	6 (0.9)	7 (2.2)	
Orthopaedic surgery-trauma	50 (4.4)	32 (5.0)	18 (5.5)	
Other	31 (2.7)	13 (2.0)	13 (4.0)	
Medical				
Cardiac	44 (3.8)	13 (2.0)	15 (4.6)	
Gastrointestinal-hepatic	5 (0.4)	4 (0.6)	4 (1.2)	
Oncologic-hematologic	11 (1.0)	0 (0.0)	5 (1.5)	
Neurologic	77 (6.7)	33 (5.1)	19 (5.8)	
Renal	1 (0.1)	0 (0.0)	1 (0.3)	
Respiratory	116 (10.1)	38 (5.9)	26 (8.0)	
Other	63 (5.5)	28 (4.3)	28 (8.6)	
Condition on admission				
Mechanical ventilation required, *N* (%)	1033 (90.0)	580 (90.1)	286 (87.7)	
ECMO or other assist device, required, *N* (%)	40 (3.5)	14 (2.2)	9 (2.8)	
Infection, *N* (%)	415 (36.1)	173 (26.9)	116 (35.6)	

Data are expressed as number (%) or median (IQR)

*STRONGkids* Screening Tool for Risk on Nutritional Status and Growth, *PeLOD* Paediatric Logistic Organ Dysfunction, *PIM2* Paediatric Index of Mortality 2, *ECMO* extracorporeal membrane oxygenation, *PICU* paediatric intensive care unit

^a^There were no significant differences in sex, age, and number of infants between matched subgroups of patients and controls

^b^Height and weight, expressed as percentiles of population norms, were calculated with the anthropometric calculators for normal children, based on the World Health Organisation Growth Charts for Canada (version 2015/02/24), and for children with syndromes known to affect height and weight (version 2014/09/25). Patients and healthy children were not matched for height and weight, as a growth delay is expected in critically ill children

^c^Dichotomizing label indicating whether or not the patient was suffering from any symptomatic chronic disease identified through screening of the patient’s medical history and hospital files

^d^Scores on the STRONGkids range from 0 to 5, with a score of 0 indicating a low risk of malnutrition, a score of 1–3 indicating medium risk, and a score of 4–5 indicating high risk

^e^PeLOD scores range from 0 to 71, with higher scores indicating more severe illness

^f^PIM2 scores, with higher scores indicating a higher risk of mortality

^g^PIM2 probability of death, ranging from 0% to 100%, with higher percentages indicating a higher probability of death in PICU

For the full study protocol, we refer to the original article and electronic supplementary protocol [2, 17]. The institutional ethical review boards of the centres in Leuven (ML8052), Rotterdam (NL38772.000.12) and Edmonton (Pro00038098) approved the study, which was performed in accordance with the 1975 Declaration of Helsinki as revised in 1983. Written informed consent for participation in the trial, blood sampling and data analyses was obtained from the parents or legal guardians.

### Leukocyte telomere length measurements

Patient blood samples were collected upon PICU admission and on the last PICU day. For comparison, blood was sampled from 342 healthy children who had never been admitted to a PICU, immediately after intravenous catheterisation prior to minor elective surgery (Fig. 1, Table 1). Relative telomere length in leukocyte DNA was quantified as telomere/single-copy-gene (T/S) ratio (proportional to average telomere length in a cell [18]) with quantitative PCR in the laboratory of Prof. Elizabeth Blackburn (Additional file 1) [19].

### Statistical analyses

Data are presented as mean and standard error (SE) or 95% confidence interval (CI), median and interquartile range (IQR), or number and proportion, as appropriate. Univariate comparisons were performed with the chi-square (Fisher exact) test for proportions, and with the Wilcoxon rank-sum or Student *t* test depending on the type of distribution of continuous data.

For *comparing leukocyte telomere lengths of critically ill patients with those of healthy children*, given that age, sex, and environment affect telomere length [20, 21], we selected demographically comparable cohorts of patients and controls via propensity-score matching with age, sex and treatment centre as covariates, yielding 326 critically ill and 326 healthy children (Fig. 1, Table 1, Additional file 1).

The impact of *early PN, as compared with late PN, on the change in leukocyte telomere length from admission to the last PICU day* was first assessed in a *univariate way* for the total number of patients for whom leukocyte telomere length was determined (*N* = 1148). This was followed by *multivariable linear regression analysis* of the impact of randomisation to early PN or late PN, in the subset of patients for whom neutrophil counts were available (*N* = 644). This was necessary to allow adjustment for the change in neutrophil fraction from admission to last PICU day, given that neutrophils have shorter telomeres than lymphocytes [22, 23]. This multivariable linear regression analysis of the impact of randomisation to early PN or late PN was further adjusted for (a) other determinants of telomere length, being age (as telomeres shorten with age) [21], gender (with shorter telomeres expected for boys [20]) and the telomere length upon admission, (b) the *acute critical-illness*-related baseline risk factors, being the admission diagnosis, the degree of organ failure (Paediatric Logistic Organ Dysfunction (PeLOD) score), the estimated risk of death (Paediatric Index of Mortality 2 (PIM2) score), the risk of malnutrition (Screening Tool for Risk on Nutritional Status and Growth (STRONGkids) risk group), whether or not there was an infection present upon admission and the treatment centre and (c) for markers of *pre-existing chronic* disease (height and weight as percentiles of population norms) and “chronicity” as a dichotomised label indicating whether or not the patient was suffering from any symptomatic chronic disease identified via screening of the medical history and hospital files (Additional file 1), with telomeres expected to be shorter in patients with chronic illnesses [7, 10, 11, 20, 24]. To also investigate whether any impact of early PN versus late PN on telomere length change over time in PICU could be *mediated by its negative effect on time to recovery*, the multivariable linear regression analysis of the impact of randomisation to early PN or late PN was further adjusted for the duration of PICU stay. *Finally*, as a *sensitivity analysis*, we further adjusted the model for *post-randomisation factors affected by early versus late PN* that could potentially be associated with telomere length attrition. These included exposure to higher cumulative doses of insulin and a higher risk of developing new infections during the course of PICU stay [2, 6, 25]. Given the collinearity between PICU duration of stay and these post-randomisation effects of early PN, the PICU stay was replaced by these factors in the model.

Statistical analyses were performed with JMP®12.0.0 (SAS Institute, Inc., Cary, NC, USA). Two-sided *P* values of 0.05 or lower were considered statistically significant. No corrections for multiple comparisons were done.

## Results

The propensity-score matched comparison revealed that median (IQR) leukocyte telomere length in critically ill children upon PICU admission (1.625 (1.446–1.825)) was *6% shorter than that in healthy children* (1.727 (1.547–1.915)) (*P* < 0.0001).

**Fig. 2:**
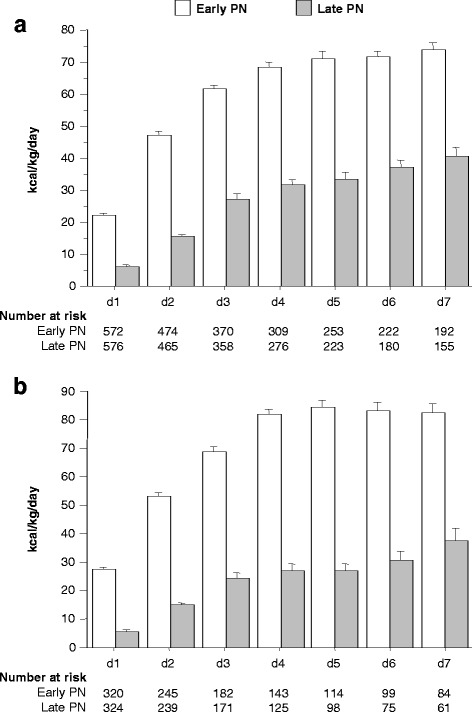
Daily caloric intake of patients in the early parental nutrition (PN) and late PN groups. The total caloric intake per day of patients in the early and late PN groups was calculated for the first week in the paediatric ICU (PICU) for the total number of patients for whom leukocyte telomere length was determined (*N* = 1148) (**a**), and for the subset of patients for whom neutrophil counts were available (*N* = 644) (**b**), with N indicating the number of patients for whom these data were available. Data are presented as mean and standard error of the mean (SEM). d, day; kcal, kilocalories

The patients in the *early PN and late PN groups* had comparable baseline characteristics (Additional file 1: Tables S1 and S2). The amount of nutrition given to patients in the early PN and late PN groups is depicted in Fig. 2.

**Fig. 3:**
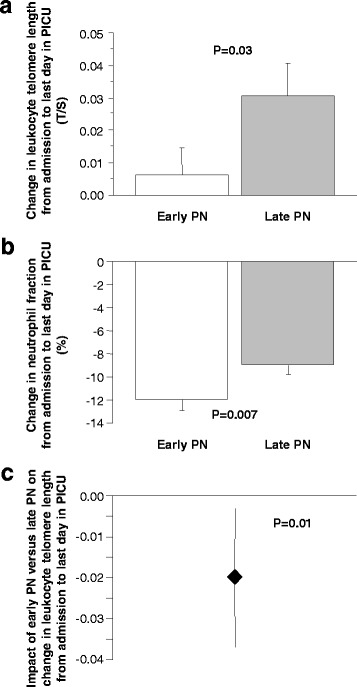
Illustration of the impact of the randomised nutritional management on the change in leukocyte telomere length. Differences were calculated between telomere length on the last day in the paediatric ICU (PICU) and that upon admission to the PICU (**a**), and between neutrophil fraction on the last day in PICU and that upon admission to the PICU (**b**), and the impact of the randomised intervention on these changes was calculated in univariate analysis. Data are presented as mean and standard error of the mean (SEM). Adjusted estimates and corresponding 95% confidence intervals were calculated for the telomere-shortening effect of early versus late PN (**c**). PN, parenteral nutrition; T/S, telomere/single-copy-gene ratio

We first assessed the impact of the randomised nutritional management on the change in telomere length during PICU stay in a univariate analysis. The change in overall leukocyte telomere length from PICU admission towards PICU discharge was significantly different between patients randomised to early PN and patients randomised to late PN (*P* = 0.03) (Fig. 3a). Indeed, whereas an apparent increase was observed in patients randomised to late PN, this rise was abolished by early PN. Importantly, neutrophils are known to have shorter telomeres than lymphocytes [22, 23], which could confound the analysis if the neutrophil fraction changed within the same time window. The neutrophil fraction decreased from PICU admission to discharge in both groups, but even more in the early PN than in the late PN group (*P* = 0.007) (Fig. 3b). Thus, the leukocyte pool in patients receiving early PN relatively shifted towards a population containing fewer cells, which under baseline conditions would have shorter telomeres (i.e. neutrophils) as compared with late PN. In combination, these findings suggest that early PN actually shortened leukocyte telomeres during PICU stay as compared with late PN.

As several other factors, apart from the leukocyte composition, have been identified that could determine telomere length and hence could introduce bias, we subsequently investigated the impact of early PN versus late PN on the change in telomere length from admission to the last day in the PICU, adjusted for these factors in multivariable regression analyses. Also when fully adjusting for potential baseline confounders and change in neutrophil fraction (see “Methods”), *early PN was significantly associated with shortened telomeres during the time in the PICU* as compared with late PN (estimate for early PN versus late PN –0.021 T/S units, 95% CI –0.038; –0.004, *P* = 0.01) (Table 2, A; Fig. 3c). The effect size of the shortening of telomere length by early PN with 0.021 T/S units represents 1.1% of the telomere length in the healthy children. Other independent determinants of change in telomere length identified in this model were age, gender, baseline telomere length and fraction of neutrophils in the sample from which the DNA was extracted (Table 2, A).

**Table 2:** Multivariable linear regression analyses determining significant and independent associations between risk factors and the change in leukocyte telomere length from admission to the last day in PICU

	A		B		C	
Variable	Estimate (95% CI) for change in telomere length expressed as T/S units, adjusted for risk factors	*P* value	Estimate (95% CI) for change in telomere length expressed as T/S units, adjusted for risk factors, including duration of PICU stay	*P* value	Estimate (95% CI) for change in telomere length expressed as T/S units, adjusted for risk factors, including cumulative dose of insulin and acquisition of new infection during PICU stay	*P* value
Randomisation to early vs. late initiation of PN	*−0.021 (−0.038; –0.004)*	*0.01*	*−0.020 (−0.037;–0.003)*	*0.01*	*−0.018 (−0.035; –0.0001)*	*0.04*
Change in fraction of neutrophils from admission to the last day (per % added)^a^	*−0.106 (−0.165; –0.048)*	*0.0004*	*− 0.115 (−0.174; –0.056)*	*0.002*	*−0.103 (−0.162; –0.044)*	*0.0006*
Age (per year added)	*−0.073 (−0.112; –0.034)*	*0.0003*	*−0.075 (−0.114; –0.037)*	*0.0002*	*−0.075 (−0.114; –0.036)*	*0.0002*
Male vs. female sex	*−0.017 (−0.034; 0.000)*	*0.05*	−0.016 (−0.033; 0.001)	0.06	*−0.016 (−0.033; 0.000)*	*0.05*
Leukocyte telomere length upon admission (per T/S unit added)	*−0.290 (− 0.356; –0.224)*	*< 0.0001*	*−0.290 (−0.356; –0.225)*	*< 0.0001*	*−0.288 (− 0.353; –0.222)*	*< 0.0001*
Diagnostic category (as compared with all other categories)						
Surgical						
Abdominal	*0.138 (0.022; 0.253)*	*0.01*	*0.135 (0.019; 0.250)*	*0.02*	*0.135 (0.019; 0.250)*	*0.02*
Cardiac	−0.012 (−0.059; 0.036)	0.63	−0.016 (−0.065; 0.032)	0.50	−0.009 (−0.057; 0.039)	0.71
Neurosurgery-traumatic brain injury	−0.025 (−0.092; 0.042)	0.45	−0.021 (−0.088; 0.046)	0.54	−0.023 (−0.090; 0.045)	0.51
Thoracic	0.008 (−0.073; 0.088)	0.85	0.007 (−0.073; 0.087)	0.86	0.004 (−0.077; 0.084)	0.93
Transplantation	−0.051 (−0.211; 0.109)	0.53	−0.060 (−0.220; 0.100)	0.45	−0.055 (−0.215; 0.105)	0.49
Orthopaedic surgery-trauma	0.022 (−0.062; 0.106)	0.60	0.026 (−0.058; 0.109)	0.54	0.029 (−0.055; 0.113)	0.50
Other	0.091 (−0.019; 0.202)	0.10	0.090 (−0.020; 0.201)	0.10	0.094 (−0.016; 0.205)	0.09
Medical						
Cardiac	−0.048 (−0.185; 0.089)	0.49	−0.050 (−0.187; 0.087)	0.47	−0.040 (−0.178; 0.097)	0.56
Gastrointestinal-hepatic	−0.091 (−0.294; 0.112)	0.37	−0.098 (−0.300; 0.104)	0.34	−0.088 (−0.291; 0.114)	0.39
Neurologic	−0.012 (−0.091; 0.067)	0.76	−0.016 (−0.095; 0.063)	0.69	−0.017 (−0.096; 0.063)	0.67
Respiratory	−0.014 (−0.113; 0.085)	0.78	−0.011 (−0.110; 0.088)	0.82	−0.024 (−0.123; 0.076)	0.64
Other	−0.006 (−0.116; 0.105)	0.91	0.015 (−0.097; 0.127)	0.79	−0.006 (−0.116; 0.105)	0.92
PeLOD score first 24 h (per point added)^b^	−0.020 (−0.069; 0.028)	0.40	−0.015 (−0.064; 0.034)	0.54	−0.019 (−0.068; 0.030)	0.44
PIM2 score (per point added)^c,d^	0.031 (−0.048; 0.111)	0.44	0.048 (−0.033; 0.128)	0.24	0.038 (−0.043; 0.120)	0.35
High vs. medium STRONGkids risk level^e^	−0.015 (−0.052; 0.022)	0.42	0.013 (−0.024; 0.050)	0.49	0.013 (−0.024; 0.051)	0.47
Infection vs. no infection upon-admission	−0.009 (−0.033; 0.015)	0.46	−0.004 (−0.029; 0.021)	0.74	−0.004 (−0.029; 0.021)	0.76
Centre (Leuven vs. Rotterdam)	0.084 (−0.030; 0.198)	0.14	0.090 (−0.024; 0.204)	0.12	0.087 (−0.027; 0.201)	0.13
Height (per percentile of population norms added)^f^	0.006 (−0.029; 0.041)	0.72	0.005 (−0.030; 0.039)	0.79	0.005 (−0.029; 0.040)	0.76
Weight (per percentile of population norms added)^f^	0.005 (−0.032; 0.042)	0.79	0.006 (−0.031; 0.043)	0.75	0.007 (−0.030; 0.044)	0.72
Chronicity vs. no chronicity^g^	0.020 (−0.009; 0.050)	0.17	0.025 (−0.054; 0.005)	0.09	0.021 (−0.008; 0.051)	0.15
Duration of stay in the PICU (per day added)			*−0.144 (−0.284; –0.004)*	*0.04*		
Cumulative dose of insulin (per IU/kg added)					−0.091 (−0.198; 0.017)	0.09
New infection vs. no new infection					0.024 (−0.012; 0.059)	0.19

*Abbreviations: PeLOD* Paediatric Logistic Organ Dysfunction, *PIM2* Paediatric Index of Mortality 2, *PICU* paediatric ICU, T/S telomere/single-gene-copy ratio, STRONGkids, Screening Tool for Risk on Nutritional Status and Growth, *IU* international units

Independent determinants of change in telomere length are indicated in italics

^a^The fraction of neutrophils is the percentage neutrophils of the total leukocyte count, ranging from 0 to 100%.

Analyses were performed in 508 patients for whom all variables were available

^b^PeLOD scores range from 0 to 71, with higher scores indicating more severe illness

^c^PIM2 scores, with higher scores indicating a higher risk of mortality

^d^Including the PIM2 probabilities of death (ranging from 0% to 100%, with higher percentages indicating a higher probability of death in PICU) instead of the PIM2 scores, did not change any of the results. Per % added, the estimate expressed as T/S units (95% CI) for PIM2 probability of death, was 0.0002 (−0.0005; 0.0009), *P* = 0.63. In the model adjusted for duration of stay in the PICU, the estimate expressed as T/S units (95% CI) per % PIM2 probability of death added was 0.0003 (−0.0004; 0.001), *P* = 0.41

^e^Scores on the STRONGkids range from 0 to 5, with a score of 0 indicating a low risk of malnutrition, a score of 1–3 indicating medium risk, and a score of 4–5 indicating high risk

^f^Height and weight, expressed as percentiles of population norms, were calculated with the anthropometric calculators for normal children, based on the World Health Organisation Growth Charts for Canada (version 2015/02/24), and for children with syndromes known to affect height and weight (version 2014/09/25)

^g^Dichotomising label indicating whether or not the patient was suffering from any symptomatic chronic disease identified through screening of the patient’s medical history and hospital files

Further adjusting the model for duration of PICU stay revealed that the telomere-shortening effect of early PN was largely independent of its impact on time to recovery. Indeed, although a longer PICU stay was associated with telomere shortening (estimate per day in PICU –0.144 T/S units, 95% CI −0.284; –0.004, *P* = 0.04), the use of early PN remained independently associated with shortened telomeres on the last PICU day (estimate of early PN versus late PN –0.020 T/S units, 95% CI −0.037; –0.003, *P* = 0.01) in this model, as did age, baseline telomere length, and change in fraction of neutrophils (Table 2, B).

Finally, the sensitivity analysis, in which we further corrected the multivariable analysis for post-randomisation factors affected by early versus late PN, confirmed the robustness of the initial findings (Table 2, C). Indeed, early initiation of PN remained independently associated with telomere length attrition (estimate of early PN versus late PN –0.018 T/S units, 95% CI −0.035; –0.0001, *P* = 0.04), together with age, gender, baseline telomere length, and change in fraction of neutrophils, whereas the amount of insulin given and the acquisition of a new infection in PICU were not related to the change in telomere length.

## Discussion

Critically ill children presented to the PICU with significantly shorter leukocyte telomeres than matched healthy children. More importantly, early initiation of PN during critical illness, as compared with postponing PN to beyond the first week in PICU, was associated with shortened leukocyte telomeres between PICU admission and discharge. The telomere-shortening effect of early PN was a robust finding, as it was maintained after adjustment for other risk factors. Independent of the telomere-shortening effect of early PN, children with a longer duration of stay in the PICU also had shortened leukocyte telomere length at PICU discharge.

In line with studies showing that children suffering from chronic illnesses have shorter telomeres than healthy peers [10, 11], we found that critically ill children have shorter leukocyte telomeres upon admission to the PICU than matched controls. In theory, this could (partly) be explained by underlying chronic diseases hallmarked by hypoxemia or inflammation, which could predispose to adverse outcomes, by previous hospital admissions or by unknown differences in environmental exposures [12, 26]. Indeed, 82.6% of the patients in our study cohort suffered from chronic comorbidity. In addition, another partial explanation for the shorter telomeres upon PICU admission could be the acute rise in the fraction of neutrophils within the white blood cell population of critically ill patients, as neutrophils have shorter telomeres than lymphocytes [27, 28].

The observed 6% shorter telomeres in patients admitted to PICU represents a large difference with healthy children. Indeed, in other paediatric settings, differences of only 0.5–4% have been reported [9–11]. For example, in children with alpha1-antitrypsin deficiency, 2% shorter leukocyte telomeres have been related to high levels of the oxidative stress biomarkers, malonyldialdehyde, 8-hydroxydeoxyguanosine and H_2_O_2_ [10]. Furthermore, 4% shorter telomeres have been reported in adolescents who were born extremely premature, and this was found to be related to the respiratory dysfunction in these children [11]. This may suggest that even small differences in telomere length may potentially lead to clinically relevant alterations in phenotype.

The early use of PN, as compared with not using PN and accepting a large macronutrient deficit that often occurs when only relying on enteral feeding during the first week in PICU, was found to further shorten telomeres from PICU admission to discharge. This effect also remained robust after adjustment for potential confounders comprising, among other risk factors, type and severity of illness, admission telomere length and change in neutrophil fraction. The effect size of the early PN-induced telomere shortening determined in the adjusted model represented 1.1% of the normal telomere length, which, although smaller than the difference between patients and healthy children, was still in the order of magnitude evoked by other environmental factors [15, 29]. The early PN effect extended beyond its negative impact on intensive care dependency, as both use of early PN and longer PICU stay were independently associated with telomere shortening from PICU admission to discharge. The early PN effect on telomere length was also independent of other post-randomisation factors affected by early PN, including the larger amount of insulin given and the higher risk of acquiring a new infection in the PICU. A telomere shortening effect of early PN is in line with the results from previous studies in rats that showed slower telomere shortening with caloric restriction than with ad libitum feeding [14]. Also large population-based human studies showed that caloric restriction and better adherence to a healthy diet were independently associated with better preserved telomere length over time [15, 30]. The mechanisms explaining attenuation of telomere attrition via caloric restriction remain speculative, but have been suggested to involve anti-oxidative, anti-inflammatory or other stress-mediated pathways [6, 15]. Also, autophagy could be involved. Indeed, autophagy preserves the regenerative capacity of stem cells [31] and caloric restriction is a powerful activator of autophagy [32]. Furthermore, pharmacological autophagy activation has shown to induce telomerase activity [33].

In line with previous reports in non-critically ill patients we found that, besides early initiation of PN and duration of critical illness, age, male gender and initial telomere length are also determining factors of telomere length shortening (Table 2-B). Indeed, in most somatic cells telomere length declines with age, with a most pronounced loss during early life, which can be explained by steady proliferation of stem cells and immune cells after birth [21]. After infancy, a deceleration in telomere attrition is observed, most likely reflecting an intrinsic, ontogeny-related change in stem cell turnover and function. Furthermore, illnesses of all kind have also repeatedly been associated with more cell replication cycles in immune cells, which could further shorten telomeres [10, 12]. With regard to gender, the existing evidence is consistent in reporting longer leukocyte telomeres in women than in men [20, 34]. This has been attributed to a slower rate of telomere attrition in women, possibly due to higher oestrogen levels leading to more optimal metabolising of reactive oxygen species [35]. In contrast to previous studies in the setting of diabetes mellitus and infectious disease [25, 36, 37], the amount of insulin given and the acquisition of a new infection were not independently associated with telomere shortening in our study.

Shorter telomeres have been associated with long-term health issues, such as poor lung function, risk of cancer, cognitive decline and poor metabolic health [11, 12, 38–40]. Children who have been treated in intensive care suffer from a substantial long-term legacy of that critical illness, characterised by a physical and neurocognitive developmental impairment, as documented 4–7 years after PICU admission [3, 41]. The shorter telomeres upon PICU admission could suggest that part of this long-term legacy is explained by their risk profile upon admission. However, as the duration of PICU stay and the use of early PN were found to be associated with further telomere shortening, one could hypothesise that the stress of the illness and/or the treatments in PICU could add to the long-term legacy. Currently, involvement of accelerated telomere attrition in that long-term legacy has not been investigated. However, the telomere shortening effect of an extended PICU stay and of early PN administration could in theory consume part of the “telomeric reserve” of these children, which could make them prone to chronic conditions such as asthma, recurrent and/or chronic viral infections and neurocognitive impairment [11, 39, 42]. Hence, identifying measures to attenuate telomere shortening in PICU could potentially bring about important long-term benefit to the children. As our study suggests an iatrogenic telomere shortening effect of early PN and thus potential long-term harm, while the PEPaNIC study has shown lack of short-term benefit with an actual increased risk of new infection and delayed recovery with early PN [2], this may further support withholding of early PN in critically ill children. However, the results of an ongoing, extensive medical and neurocognitive long-term follow up of the PEPaNIC study cohort are awaited, which will allow investigation of the actual clinical relevance of the observed telomere shortening with longer PICU stays and with the use of early PN, as a biomarker or a mediator of susceptibility to long-term adverse outcomes after paediatric critical illnesses.

The strengths of this study comprise the multicentre setting of the PEPaNIC randomized controlled trial, the large group of healthy children and the paired measurements per patient allowing documentation of leukocyte telomere length dynamics from PICU admission to discharge within the same child. Importantly, the telomere shortening effect of early PN was found in the context of a large randomised controlled study design and could be confirmed after full adjustment for known potential confounders.

Some limitations should also be considered. Indeed, leukocyte composition was only available in a subgroup of patients and did not differentiate between e.g. lymphocyte subtypes. However, the subpopulation of patients in whom neutrophil counts were available was large and representative of the total population. Also, although we identified factors that may contribute to accelerated telomere shortening during the course of the PICU stay, the long-term outcome of these children should be documented to investigate whether shorter telomeres partially explain the observed legacy following paediatric critical illness.

## Conclusions

Shorter-than-normal leukocyte telomeres were observed in children upon PICU admission. Strikingly, early initiation of PN during critical illness and an extended duration of PICU stay further shortened leukocyte telomeres between PICU admission and discharge. Whether this predisposes to long-term consequences of paediatric critical illness and “unhealthy ageing” remains to be investigated in a prospective follow-up study.

## Additional file

Additional file 1: Leukocyte telomere length measurements. Propensity score matching. Definition of “chronicity” as a dichotomising label indicating whether the patient was suffering from any symptomatic disease. **Table S1.** Demographics of patients randomised to early versus late initiation of parenteral nutrition (PN) for the total number of patients for whom leukocyte telomere length was determined (*N* = 1148). **Table S2.** Demographics of patients randomised to early versus late initiation of parenteral nutrition (PN) for the subset of patients for whom neutrophil counts were available (*N* = 644). References. (DOC 126 kb)

## Abbreviations

ECMO: Extracorporeal membrane oxygenation
IQR: Interquartile range
PCR: Polymerase chain reaction
PeLOD score: Paediatric Logistic Organ Dysfunction score
PEPaNIC-study: Paediatric Early versus late Parenteral Nutrition in Intensive Care unit study
PICU: Paediatric intensive care unit
PIM2 score: Paediatric Index of Mortality 2 score
PN: Parenteral nutrition
RCT: Randomised controlled trial
STRONGkids: Screening Tool for Risk on Nutritional Status and Growth
T/S ratio: Telomere/single-copy-gene ratio
