# Supplementary material for: Leukocyte telomere length in paediatric critical illness: effect of early parenteral nutrition
Source: Crit Care. 2018 Feb 21;22:38. doi: 10.1186/s13054-018-1972-6 (PMC5820800; doi:10.1186/s13054-018-1972-6)
Supplement: Supplementary file 1 — Leukocyte telomere length measurements. Propensity score matching. Definition of “chronicity” as a dichotomising label indicating whether the patient was suffering from any symptomatic disease. Table S1. Demographics of patients randomised to early versus late initiation of parenteral nutrition (PN) for the total number of patients for whom leukocyte telomere length was determined (N = 1148). Table S2. Demographics of patients randomised to early versus late initiation of parenteral nutrition (PN) for the subset of patients for whom neutrophil counts were available (N = 644). References. (DOC 126 kb) [file 13054_2018_1972_MOESM1_ESM.doc]

**Additional file 1**

**Leukocyte telomere length in paediatric critical illness:**

**effect of early parenteral nutrition**

Sören Verstraete, M.D.*^1^, Ilse Vanhorebeek, Ph.D.*^1^, Esther van Puffelen, M.D.²,

Inge Derese, B.Sc.^1^, Catherine Ingels, M.D., Ph.D.^1^, Sascha C Verbruggen, M.D., Ph.D.², Pieter J Wouters, M.Sc.^1^, Koen F Joosten, M.D., Ph.D.², Jan Hanot, M.D.^1^,

Gonzalo G Guerra, M.D.³, Dirk Vlasselaers, M.D., Ph.D.^1^, Jue Lin, Ph.D.^4^,

Greet Van den Berghe, M.D., Ph.D.^1^

**Leukocyte telomere length measurements**

**Propensity score matching**

**Definition of “chronicity” as a dichotomising label indicating whether the patient was suffering from any symptomatic chronic disease**

**Table S1: Demographics of patients randomised to early versus late initiation of parenteral nutrition (PN) for the total number of patients for whom leukocyte telomere length was determined (N=1148)**

**Table S2: Demographics of patients randomised to early versus late initiation of parenteral nutrition (PN) for the subset of patients for whom neutrophil counts were available (N=644)**

**References**

**Leukocyte telomere length measurements**

Blood samples were collected from the patients upon PICU admission, on days 3, 5 and 7, and on the last day in PICU. For comparison, blood was sampled from 342 healthy children who had never been admitted to a PICU, immediately after placement of an intravenous catheter prior to minor elective surgery (Figure_1, Table_1). Cell pellets obtained after centrifugation of the blood samples were stored at -80°C. Genomic DNA was extracted from the leukocytes in the pellets with the Maxwell®RSC instrument and corresponding blood DNA purification kit (Promega Benelux b.v., Leiden, The Netherlands). DNA concentration was quantified with a Nanodrop® ND-1000 spectrophotometer (Isogen Life Science, The Meern, The Netherlands). Samples were loaded onto 96-well plates at 30 ng/µL and DNA was stored at -80°C until shipment on dry ice to the laboratory of Prof. Elizabeth Blackburn for telomere length quantification. Relative telomere lengths [i.e., Telomere-to-Single copy gene (T/S) ratio, which is proportional to the average telomere length in a cell ] were measured by quantitative PCR . All samples were analysed in triplicate wells and run twice. When the values differed by more than 7%, the assay was run a third time and the two closest values were averaged. Average CV was 2.2% (±1.7%). The laboratory personnel was blinded to participants' characteristics and randomisation.

**Propensity score matching**

For comparing telomere lengths of critically ill patients with those of healthy children, given that age, sex, and environment affect telomere length , we first selected demographically comparable cohorts of patients and controls via propensity-score matching with the SPSS R-menu R3.1 (Foundation for Statistical Computing) in IBM SPSS Statistics 23.0.0.0 (SPSS, Chicago, IL). Logistic regression was used to estimate propensity scores with age, sex, and treatment centre as covariates. A caliper of 0.01 was used for one-to-one nearest-neighbour matching, yielding 326 critically ill and 326 healthy children (Figure 1, Table 1).

**Definition of “chronicity” as a dichotomising label indicating whether the patient was suffering from any symptomatic chronic disease**

One investigator (SV), screened the patient’s medical history and hospital files and dichotomised patients according to whether they suffered from any symptomatic “chronic” disease or not. Each condition reported as a cause of an individual’s activity limitation and/or the need for ongoing medical care has been classified as chronic. Conditions that are not cured once acquired (such as heart disease, diabetes, and birth defects) were considered chronic. Other conditions had to be present for at least 3 months or longer to be considered chronic. An exception is made for children aged younger than 1 year who have had a condition since birth, where such conditions were always considered chronic. In case of doubt, SV and GVdB jointly decided whether the patient should be labelled as “chronic” or “not chronic“.

| **Table S1: Demographics of patients randomised to early PN or late PN for the total number of patients for whom leukocyte telomere length was determined (N=1148)** | | | |
| --- | --- | --- | --- |
|  |  |  |  |
| **Baseline characteristics** | **Early PN N=572** | **Late PN N=576** | **P-value** |
|  |  |  |  |
| Male gender | 314 (54.9) | 326 (56.6) | 0.59 |
| Age (years) | 1.8 (0.3-6.7) | 1.8 (0.3-8.4) | 0.37 |
| Age < 1 year | 240 (42.0) | 236 (41.0) | 0.73 |
| Height (%)^a^ | 36.9 (6.9-78.1) | 40.1 (9.4-79.2) | 0.46 |
| Weight (%)^a^ | 31.0 (8.2-69.2) | 34.2 (8.9-66.2) | 0.56 |
| Chronicity^b^ | 445 (77.8) | 437 (75.9) | 0.44 |
| STRONGkids risk level^c^ |  |  | 0.60 |
| Medium | 524 (91.6) | 522 (90.6) |  |
| High | 48 (8.4) | 54 (9.4) |  |
| PeLOD score first 24 hrs^d^ | 22 (12-32) | 22 (12-32) | 0.81 |
| PIM2 score^e^ | -2.7 (-3.6;-1.3) | -2.7 (-3.6;-1.6) | 0.71 |
| PIM2 probability of death (%)^f^ | 6.1 (2.6-21.3) | 6.5 (2.6-17.2) | 0.72 |
| Emergency admission | 271 (47.4) | 283 (49.1) | 0.55 |
| Diagnostic category |  |  | 0.92 |
| Surgical |  |  |  |
| Abdominal | 25 (4.4) | 26 (4.4) |  |
| Burns | 3 (0.5) | 5 (0.9) |  |
| Cardiac | 263 (46.0) | 254 (44.1) |  |
| Neurosurgery-traumatic brain injury | 56 (9.8) | 50 (8.7) |  |
| Thoracic | 23 (4.0) | 23 (4.0) |  |
| Transplantation | 7 (1.2) | 15 (2.6) |  |
| Orthopaedic surgery-trauma | 26 (4.6) | 24 (4.2) |  |
| Other | 12 (2.1) | 19 (3.3) |  |
| Medical |  |  |  |
| Cardiac | 22 (3.9) | 22 (3.8) |  |
| Gastrointestinal-hepatic | 2 (0.4) | 3 (0.5) |  |
| Oncologic-hematologic | 5 (0.9) | 6 (1.0) |  |
| Neurologic | 40 (7.0) | 37 (6.4) |  |
| Renal | 0 (0.0) | 1 (0.2) |  |
| Respiratory | 56 (9.8) | 60 (10.4) |  |
| Other | 32 (5.6) | 31 (5.4) |  |
| Condition on admission |  |  |  |
| Mechanical ventilation required | 519 (90.7) | 514 (89.2) | 0.39 |
| ECMO or other assist device required | 17 (3.0) | 23 (4.0) | 0.34 |
| Infection | 213 (37.2) | 202 (35.1) | 0.44 |
|  |  |  |  |
|  | | | |
| Data are expressed as number (%) or median (IQR).  ^a^ Height and weight, expressed as percentiles of population norms, were calculated with the anthropometric calculators for normal children, based on the World Health Organization Growth Charts for Canada (version 2015/02/24), and for children with syndromes known to affect height and weight (version 2014/09/25).  ^b^ Dichotomizing label indicating whether or not the patient was suffering from any symptomatic chronic disease identified through screening of the patient’s medical history and hospital files.  ^c^ Scores on the Screening Tool for Risk on Nutritional Status and Growth (STRONGkids) range from 0 to 5, with a score of 0 indicating a low risk of malnutrition, a score of 1 to 3 indicating medium risk, and a score of 4 to 5 indicating high risk.  ^d^ Paediatric Logistic Organ Dysfunction (PeLOD) scores range from 0 to 71, with higher scores indicating more severe illness.  ^e^ Paediatric Index of Mortality 2 (PIM2) scores, with higher scores indicating a higher risk of mortality.  ^f^ Paediatric Index of Mortality 2 (PIM2) probability of death, ranging from 0% to 100%, with higher percentages indicating a higher probability of death in PICU.  Abbreviations: CI, confidence interval; ECMO, extracorporeal membrane oxygenation; PICU, paediatric intensive care unit. | | | |
| **Table S2: Demographics of patients randomised to early PN or late PN for the total number of patients for whom neutrophil counts were available (N=644)** | | | |
|  |  |  |  |
| **Baseline characteristics** | **Early PN N=320** | **Late PN N=324** | **P-value** |
|  |  |  |  |
| Male gender | 171 (53.4) | 184 (56.8) | 0.42 |
| Age (years) | 1.7 (0.3-5.8) | 1.6 (0.3-6.2) | 0.53 |
| Age < 1 year | 138 (43.1) | 138 (42.6) | 0.93 |
| Height (%)^a^ | 35.1 (5.8-70.8) | 34.9 (9.4-72.2) | 0.71 |
| Weight (%)^a^ | 30.2 (7.3-62.8) | 29.2 (6.3-61.8) | 0.64 |
| Chronicity^b^ | 267 (83.4) | 265 (81.8) | 0.60 |
| STRONGkids risk level^c^ |  |  | 0.32 |
| Medium | 304 (95.0) | 301 (92.9) |  |
| High | 16 (5.0) | 23 (7.1) |  |
| PeLOD score first 24 hrs^d^ | 31 (21-32) | 31 (21-32) | 0.35 |
| PIM2 score^e^ | -2.9 (-3.7;-1.5) | -3.0 (-3.7;-1.7) | 0.27 |
| PIM2 probability of death (%)^f^ | 5.1 (2.3-18.2) | 4.8 (2.4-15.1) | 0.27 |
| Emergency admission | 105 (32.8) | 113 (34.9) | 0.61 |
| Diagnostic category |  |  | 0.96 |
| Surgical |  |  |  |
| Abdominal | 8 (2.5) | 11 (3.4) |  |
| Burns | 0 (0.0) | 1 (0.3) |  |
| Cardiac | 191 (59.7) | 186 (57.4) |  |
| Neurosurgery-traumatic brain injury | 25 (7.8) | 26 (8.0) |  |
| Thoracic | 14 (4.4) | 15 (4.6) |  |
| Transplantation | 2 (0.6) | 4 (1.2) |  |
| Orthopaedic surgery-trauma | 16 (5.0) | 12 (3.7) |  |
| Other | 5 (1.6) | 8 (2.5) |  |
| Medical |  |  |  |
| Cardiac | 7 (2.2) | 6 (1.9) |  |
| Gastrointestinal-hepatic | 1 (0.3) | 3 (0.9) |  |
| Neurologic | 17 (5.3) | 16 (4.9) |  |
| Respiratory | 18 (5.6) | 20 (6.2) |  |
| Other | 16 (5.0) | 12 (3.7) |  |
| Condition on admission |  |  |  |
| Mechanical ventilation required | 295 (92.2) | 285 (87.7) | 0.08 |
| ECMO or other assist device required | 8 (2.5) | 6 (1.9) | 0.60 |
| Infection | 91 (28.4) | 82 (25.3) | 0.37 |
|  |  |  |  |
|  | | | |
| Data are expressed as number (%) or median (IQR).  ^a^ Height and weight, expressed as percentiles of population norms, were calculated with the anthropometric calculators for normal children, based on the World Health Organization Growth Charts for Canada (version 2015/02/24), and for children with syndromes known to affect height and weight (version 2014/09/25).  ^b^ Dichotomizing label indicating whether or not the patient was suffering from any symptomatic chronic disease identified through screening of the patient’s medical history and hospital files.  ^c^ Scores on the Screening Tool for Risk on Nutritional Status and Growth (STRONGkids) range from 0 to 5, with a score of 0 indicating a low risk of malnutrition, a score of 1 to 3 indicating medium risk, and a score of 4 to 5 indicating high risk.  ^d^ Paediatric Logistic Organ Dysfunction (PeLOD) scores range from 0 to 71, with higher scores indicating more severe illness.  ^e^ Paediatric Index of Mortality 2 (PIM2) scores, with higher scores indicating a higher risk of mortality.  ^f^ Paediatric Index of Mortality 2 (PIM2) probability of death, ranging from 0% to 100%, with higher percentages indicating a higher probability of death in PICU.  Abbreviations: CI, confidence interval; ECMO, extracorporeal membrane oxygenation; PICU, paediatric intensive care unit. | | | |

**References**

1. Cawthon RM. Telomere measurement by quantitative PCR. Nucleic Acids Res. 2002;30(10):e47.

2. Lin J, Epel E, Cheon J, Kroenke C, Sinclair E, Bigos M, Wolkowitz O, Mellon S, Blackburn E. Analyses and comparisons of telomerase activity and telomere length in human T and B cells: insights for epidemiology of telomere maintenance. J Immunol Methods. 2010;352(1-2):71-80.

3. Dalgard C, Benetos A, Verhulst S, Labat C, Kark JD, Christensen K, Kimura M, Kyvik KO, Aviv A. Leukocyte telomere length dynamics in women and men: menopause vs age effects. Int J Epidemiol. 2015;44(5):1688-1695.

4. Aubert G, Baerlocher GM, Vulto I, Poon SS, Lansdorp PM. Collapse of telomere homeostasis in hematopoietic cells caused by heterozygous mutations in telomerase genes. PLoS Genet. 2012;8(5):e1002696.
